# Maximize the use of municipal waste generated by the hydrogen peroxide industry in the production of high-quality refractory CAC

**DOI:** 10.1038/s41598-022-16891-z

**Published:** 2022-07-27

**Authors:** Ahmed I. H. Amer, A. A. El-Maddah, Ahmed M. Abuelela, Y. M. Z. Ahmed, Ali M. Hassan, Ahmed A. M. El-Amir

**Affiliations:** 1 Refractory and Ceramic Materials Department, Central Metallurgical Research & Development Institute, Cairo, Egypt; 2 Chemistry Department, Faculty of Science, Al-Azhar University, Cairo, Egypt

**Keywords:** Structural materials, Mechanical properties

## Abstract

High-grade calcium aluminate cement (CAC) has been successfully synthesized from municipal alumina waste and limestone under mild reaction conditions. Mineralogical composition and microstructure of the sintered mixes were investigated using X-ray diffraction and FESEM; valuable cementing phases such as CA, CA_2_, and C_12_A_7_ were observed in addition to the C_3_A phase that was detected in the mixes with high CaCO_3_ content. Mix CA60 containing 60 wt% alumina waste has achieved the best sinterability (less than 1 vol% porosity) and the highest densification (~ 2.65 g/cm^3^ bulk density) at 1450 °C. Densification, cold-crushing strength (CCS), and microstructure of the hydrated cement samples (From Mix CA60) were investigated. The cast cement specimens revealed better density and CCS characteristics (63.1 and 74 MPa at 7 and 28 days, respectively) in comparison with the commercial cement. Conventional castables (5 × 5 × 5 cm^3^) were prepared from mixtures composed of 15 wt% cement and 85 wt% aggregates (40% Al_2_O_3_), where CA60 and commercial cement were used to compare the effect of the manufactured CA60 cement with the commercial one. The castables prepared with CA60 cement have shown a higher strength at 110 °C with 4.5 MPa when compared to the commercial CAC at the same temperature (1.8 MPa). Accordingly, this study contributes not only to preserving the environment from the accumulation of industrial wastes but also to valorizing and adding value to these wastes.

## Introduction

Refractories are traditional ceramic materials that can afford high temperatures without deterioration. They are used in high quantities to line vessels through which the materials with high elevated temperatures are manufactured such as cement, glass, and metals. The main purposes of utilization of refractory materials are reducing the energy loss via kilns and smoothing the heat flow through the materials inside these kilns^1^. Refractory materials are composed of aggregate phases in large sizes (up to centimeters) held together with finer phases (sometimes sub-micrometers) and binder^2^. According to the physical state, refractory materials are divided into two kinds (1) unshaped refractory castables Monolithicss) and (2) shaped refractory bricks^3^. Monolithics are unfired materials with different particle sizes that are bonded together with refractory calcium aluminate cement and directly poured into the installation site.

Calcium aluminate cement (CAC) is an important hydraulic setting cement that acts as a binder for refractory castables; its use as a binder is related to its high refractoriness, and presence of excellent hydration capacity phases, and good execution in corrosive environments^4^. It consists mainly of calciummonoaluminate (CaAl_2_O_4_ (CA)) as the highest and the most important component, calciumdialuminate (CaAl_4_O_7_ (CA_2_)) that appears in lower concentration, mayenite (Ca_12_Al_14_O_33_ (C_12_A_7_)) and otnonhydrauliculic phases such as 3CaO·Al_2_O_3_ (C_3_A), CaO·6Al_2_O_3_ (CA_6_), corun, dum, and gehlenihat may appear depending on the exact ratio of CaO/Al_2_O_3_. In addition to its high melting point (around ~ 1600 °C), the CA phase produces a high mechanical strength over a short period of hydration. Even though the CA_2_ phase demonstrates a bit higher melting point (~ 1700 ℃) compared to the CA phase, the former has a long hardening time and generates low mechanical strength; hence CA_2_ can’t exist as the main component of CAC and it generally presents as a secondary phase in aluminous cement with CA. The low melting phase C_12_A_7_ (~ 1430 °C) has a rapid hardening and hydration rate and its presence as a secondary phase in the aluminous cement can easily modify and control their cementing characteristics^2,5–8^.

One of the most significant features of CAC is the development of approximately 80–90% of its final strength after just 24 h. This in turn enables its involvement in different refractory and non-refractory applications such as military installations, busy locations, and underground works^9^. Many other applications such as sewer applications^10,11^, protective coatings^10,11^, expansive grouts, and chemical building products^10,11^, have utilized CAC in combination with ordinary Portland cement (OPC) to achieve the required setting times earlier^9–13^. When compared to Portland cement annual production of CAC is quite small due to its high production cost and scarcity of alumina sources. Therefore, the utilization of calcium aluminate cement in daily applications can’t compete with Portland cement In Despite this, CAC is indispensable for specific applications in which it surpasses the performance of Portland cement. For instance, the conventional concrete produced by CAC has demonstrated an outstanding strength as well as an excellent resistance to high temperatures, abrasions, and many chemically aggressive conditions^8,14^. In addition, the production of CAC releases lower CO_2_ emissions than Portland cement production^15^.

CAC was also utilized in the remediation of heavy metals contaminated soil and has shown a better performance than OPC^16^. Calgaro et al.^16^, have investigated the performance of CAC and OPC in the treatment of contaminated soil with heavy metals (i.e. As, Ba, Be, Cu, Cd, Co, Cr, Hg, Tl, Ni, P b, Sn, Sb, Se, V, and Zn), using solidification/stabilization processes. These processes achieve encapsulation of pollutants in a solid matrix that has low levels of permeability and porosity. In addition, these pollutants were successfully transformed into less soluble, toxic, or mobile forms through the reaction with a hydraulic binder. The results have shown better performance for CAC over OPC in the immobilizations of most of the investigated metals.

The traditional raw materials utilized for CAC production are limestone as a CaO source and calcined alumina or bauxite as Al_2_O_3_ source. Industrial wastes have been used as a source of CaO and Al_2_O_3_, which is aligned with the environmental and economic trend that has been spotlighted recently^17^. For instance, a waste source of CaO is calcium hydroxide produced from the preparation of acetylene gas has been used by Zawrah et al.^9^, for the manufacture of calcium aluminate refractory cement. A purPurecommercial calcium aluminate cements were produced by sintering at 1500–1550 °C using pure calcined alumina or calcined bauxite respectively. The resulted cements have exhibited good sintering, mechanical, cementing, and refractory properties, in which the pure cement can be used at high temperatures (> 1500 °C) and the commercial one at a limited temperature (< 1500 °C).

On the other hand, the alumina sources are quite-rare and existed in a few countries besides the special processing procedure required for grinding bauxite (the main alumina source) due to its high-hardness^8,18,19^. The scarcity of alumina sources is the main reason for the high cost of aluminous cements. Thus, finding cheap alternative sources for alumina could enable CAC production at suitable and cost-effective conditions, allowing its use in many applications instead of Portland cement and giving rise to products with higher quality at suitable prices. This in turn has attracted some researchers to further investigate and modify the production of calcium aluminate cement from industrial wastes. These attempts have resulted in a high-quality CAC that can be further improved depending on the type of waste or the used additives. For instance, aluminum slag has been used as a source of Al_2_O_3_ by Ewais et al.^12^, for the manufacture of calcium aluminate cement. Aluminum sludge was also used as a source of Al_2_O_3_ and calcium oxide, in addition to pure alumina. CAC sample which was fired at 1500 °C and composed of 72.2 wt% of Al_2_O_3_, has shown the highest mechanical properties with a strength of lue ~ 50 MPa. While, the CAC sample that contains 62.55 wt% of Al_2_O_3_, has shown the highest strength value after various hydration days (1, 3, 7, and 28).

In addition, other wastes have been used in the production of alumina cement by Krivoborodovet al.^20^, as a source of alumina, such as the slag of ferroalloys manufacture and the dust of gas cleaning plants of aluminum electrolysis. The study has indicated an improvement in the properties of alumina cement related to the slag from ferrovanadium production which contains 67.4 wt% of Al_2_O_3_.

Lopez-Delgado et al.^21^, have achieved the synthesis of calcium aluminate (CA) from a Spanish hazardous waste, that has been used as a source of Al_2_O_3_. An amorphous precursor was obtained through the hydrothermal method, where the precipitation of calcium aluminate (CA) was occurring starting from 700 °C. The study has detected the transformation of the precursor in a crystalline aluminate, where the C_12_A_7_ phase was formed first and then transitioned to CA_2_ at 838–848 °C. This was followed by a transformation to CA at 1000–1034 °C.

Engbert et al.^22^, have investigated the effect of sand as a filler on the hydration of CAC which has resulted in acceleration of the hydration. Other fine filler materials such as fine limestone powder, micro silica, and α- and γ-Al_2_O_3_ have also accelerated the hydration of CAC. The detected mechanism of the acceleration was subjected to the negative surface charge that affects calcium ions under alkaline conditions.

Idrees et al.^23^, have tested the effect of mineral admixtures such as fly ash, granulated ground blast furnace slag, and silica fume on the properties of CAC mortars at different curing temperatures. It was found that the hydration reactions of CAC mortars were accelerated by using the mineral admixtures as sand replacement materials. Better mechanical strength at 20 °C was detected by using 10% of fly ash and granulated ground blast furnace slag as replacement materials for sand. CAC has been interfered with in the manufacture of new cementitious materials by Arbi et al.^24^. These materials were produced through alkaline activation of diatomite (mainly contains Si) or blast furnace slag (mainly contains Ca and Si), in the presence of CAC as a source of reactive aluminum. The obtained cementitious materials with 20% CAC and 80% blast furnace slag, have shown the highest mechanical strength value with 8 M of NaOH as an alkaline activator. The cementitious properties of the products were assigned to the formed gels whose nature (C–A–S–H– or (N, C)–A–S–H-like) depended on the activator and the prime materials used.

In Egypt, huge quantities of alumina wastes are produced annually from the hydrogen peroxide industry at El-Nasr Co. for Intermediate Chemical (NCIC) (around 240 Metric Ton). These wastes consist of pure alumina mixed with organic matter. The utilization of these wastes in the manufacture of calcium aluminate cement can achieve a huge step in the economic and environmental progress, especially with a simple and low-cost production method. Hence, in this work, high-grade CAC has been successfully produced for the first time from these alumina wastes and limestone using a facile solid-state process, achieving a double economic and environmental value via disposing of these wastes and producing high-grade aluminous cement with a super-fast setting time feature.

## Materials and methods

### Materials

Alumina industrial waste was supplied by the hydrogen peroxide factory of El-Nasr Co. for Intermediate Chemical (NCIC), Egypt. This factory produces annually around ~ 240 Metric Ton of alumina waste. It accumulates in landfill sites, creating a serious environmental problem. As shown in Table 1, this waste consisted primarily of Al_2_O_3_ (55.6 wt%) with low levels of impurities (2.5 wt%) and 41.9 wt% loss on ignition. On the other hand, limestone was supplied by the quarries of Minya, Minya, Egypt. It consisted mainly of CaCO_3_ (98.61 wt%) with minor amounts of MgO (0.75 wt%) and SiO_2_ (0.234 wt%) impurities (Table 1). Commercial cement with 50% alumina was used to be compared with the prepared one. The feedstock materials have been used in this work without any pre-treatment.

**Table 1:** Chemical analyses of starting materials.

Oxides	Chemical composition, wt%	
	Industrial Waste	Limestone
Na_2_O	0.201	–
Al_2_O_3_	55.6	0.161
SiO_2_	0.185	0.284
P_2_O_5_	1.60	–
SO_3_	0.369	0.026
CaO	0.098	55.25
Fe_2_O_3_	0.036	0.056
SrO	–	0.061
MgO	–	0.795
LOI	41.9	43.36

### Methods

Four batch compositions have been designed from the feedstock materials with 40–70 wt% alumina waste and 60–30 wt% limestone. These batches were numbered CA40-70 [CA stands for calcium aluminate and the number indicates alumina waste wt% in the mix]. The nominal compositions of the designed batch compositions have been set out in Table 2. In a typical procedure, the batches were dry-ground using a planetary ball mill at 350 rpm for 30 min to homogenize the ingredients of the blends. Thereafter, ground mixes were screened through 40-mesh sieves and shaped into cylindrical specimens via uni-axial press at 100 MPa. The shaped discs were then sintered at 1250–1450 °C for 4 h with an interval of 100 °C and a heating rate of 5 °C/min. The range of temperatures was chosen based on the phase diagram of the calcium aluminate cement^25^. Based on the phase composition and densification characteristics of the sintered specimens, the CA60 sintered specimen at 1450 °C was selected to be cast as a rapid-setting refractory cement. Thereupon, 2 kg of CA60 specimens sintered at 1450 °C were ground, mixed with water, and cast as a cement mortar in 5 × 5 × 5 cm^3^ steel molds. The hardened cement cubes were demolded after 24 h and subsequently hydrated in a humid atmosphere for 1, 3, 7, and 28 days. Unshaped conventional castable was manufactured from 15 wt% CA60 cement along with heavy, and lightweight calcined kaolin aggregates to study the binding performance of the fabricated CA60 cement in the unshaped castable. A similar castable structure was prepared using commercial calcium aluminate cement to compare the binding capability of CA60 and commercial cement in the unshaped monolithics.

**Table 2:** Nominal compositions of the designed batches.

Mix no.	Composition, wt%		Al_2_O_3_/CaO, wt%
	Alumina waste	Limestone	
CA40	40	60	0.67
CA50	50	50	1.0
CA60	60	40	1.5
CA70	70	30	2.34

### Characterization techniques

Chemical analyses of raw materials and sintered samples have been detected using the PanalyticalXRF (Model advanced Axios, Netherlands) machine. The phase composition of the sintered and hydrated specimens was detected by the powder X-ray diffractometer [Philips PW 1710 X-ray diffractometer with Cu k_α_ radiation (λ = 1.54 nm) at 40 kV, 30 mA and a scanning speed of 2°/min]. Densification parameters of the sintered and hydrated samples have been calculated using the vacuum pressure method according to ASTM C830. The microstructure of the polished surfaces of the CA60 specimens sintered at 1450 °C and hydrated for 1, 3, and 7 days have been investigated by the backscattered electron (BSE) in the FESEM (QUANTAFEG250, Holland), connected with an energy dispersive X-ray microanalyzer (EDX). Cementing properties of the cast cement mortars in terms of water of consistency, and initial and final setting time was measured using the Vicat apparatus. Cold crushing strength of both sintered and hydrated specimens was measured at a rate of 1.3 mm/min via the Shimadzu universal testing machine of compression (UH-F 1000 KN, Japan).

## Results and discussion

### Sintered samples

#### Phase composition evolution

**Figure 1:**
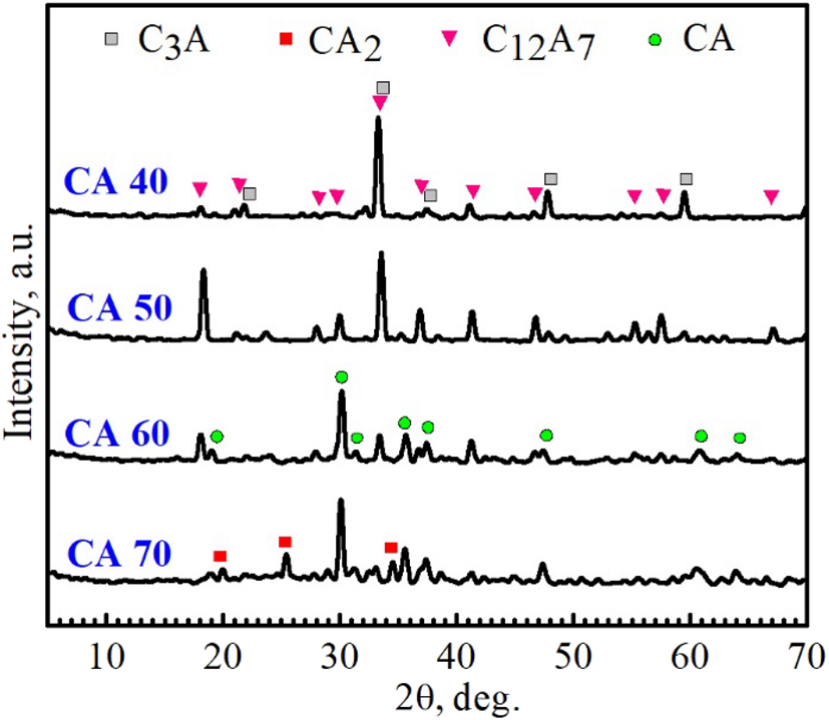
Powder XRD patterns of the compacted specimens sintered at 1250 °C.

**Figure 2:**
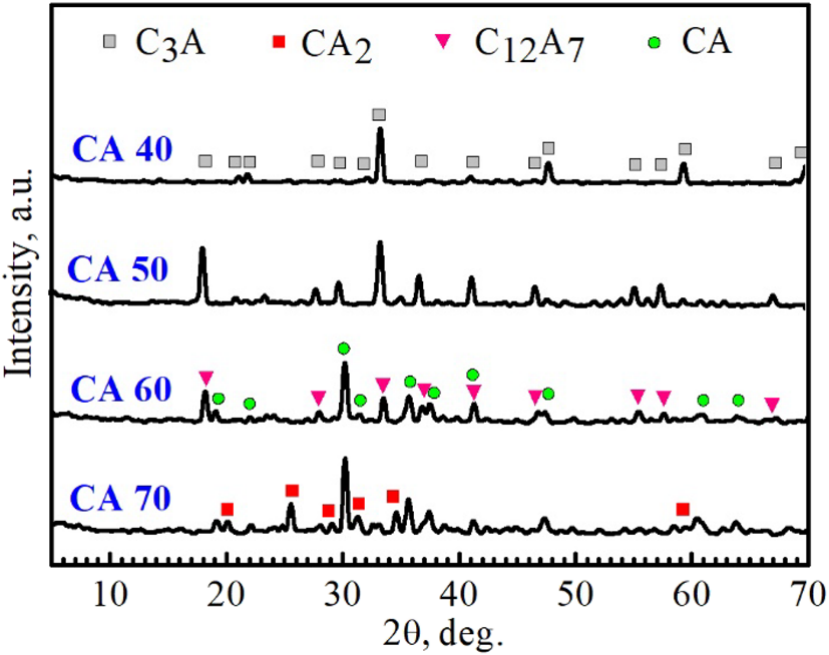
Powder XRD patterns of the compacted specimens sintered at 1350 °C.

**Figure 3:**
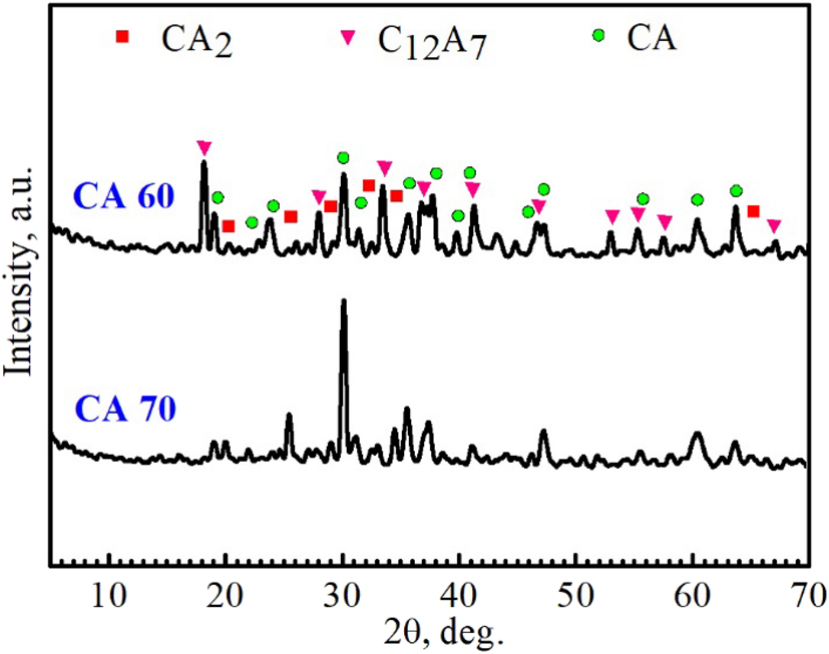
Powder XRD patterns of the compacted specimens sintered at 1450 °C.

Figures 1, 2 and 3 revealed X-ray diffraction patterns of the CA40-70 cement mixes sintered at 1250, 1350, and 1450 °C, respectively. As shown in Fig. 1, a majority of tricalcium aluminate (C_3_A) along with a minority of mayenite (C_12_A_7_) were detected in CA40 and CA50 mixes sintered at 1250 ℃. With the increase in alumina content in the designed batches, a majority of calcium monoaluminate (CA) along with a minority of mayenite and calcium aluminate (CA_2_) phases were detected in the CA60 and CA70 mixes, respectively. With the increase of sintering temperature up to 1350 °C, CA40 and CA50 mixes showed a single-phase composition of C_3_A whereas CA60 and CA70 mixes had the same structure as at 1250 °C with a majority of CA and a minority of C_12_A_7_ and CA_2_, respectively (Fig. 2). The dominance of the C_3_A phase over the mayenite phase in the case of CA40 and CA50 mixes may return to the observed increase of CaO wt% compared to the Al_2_O_3_ content in these two mixtures^26^. At 1450 °C, CA40 and CA50 mixes were fully melted, while CA60 and CA70 mixes survived due to their high alumina content. At 1450 °C, the CA60 cement blend revealed a double-phase structure with equal amounts of C_12_A_7_ and CA phases, while the CA70 cement mix had the same structure as at 1250–1350 °C with a majority of CA and a minority of CA_2_ (Fig. 3). The main hydraulic phase of calcium aluminate cement is the calcium monoaluminate phase. The appearance of a quick-setting mayenite phase in an amount similar to the main hydraulic phase in the CA60 cement mix indicates the great importance of this blend for the construction of an outstanding cement structure with superior mechanical properties and quick installation features. The crystalline phases detected in the sintered specimens were summarized in Table 3.

**Table 3:** Identified phases in the sintered specimens at 1250–1450 ℃.

Firing temperature (°C)	Specimen code	Detected phases	Card no.
1250	CA40	C_3_A	38-1429
		C_12_A_7_	09-0413
	CA50	C_3_A, C_12_A_7_	
	CA60	CA	53-0191
		C_12_A_7_	
	CA70	CA	
		CA_2_	23-1037
1350	CA40	C_3_A	
	CA50	C_3_A	
	CA60	CA, C_12_A_7_	
	CA70	CA, CA_2_	
1450	CA60	CA, C_12_A_7_	
	CA70	CA, CA_2_	

#### Densification parameters

**Figure 4:**
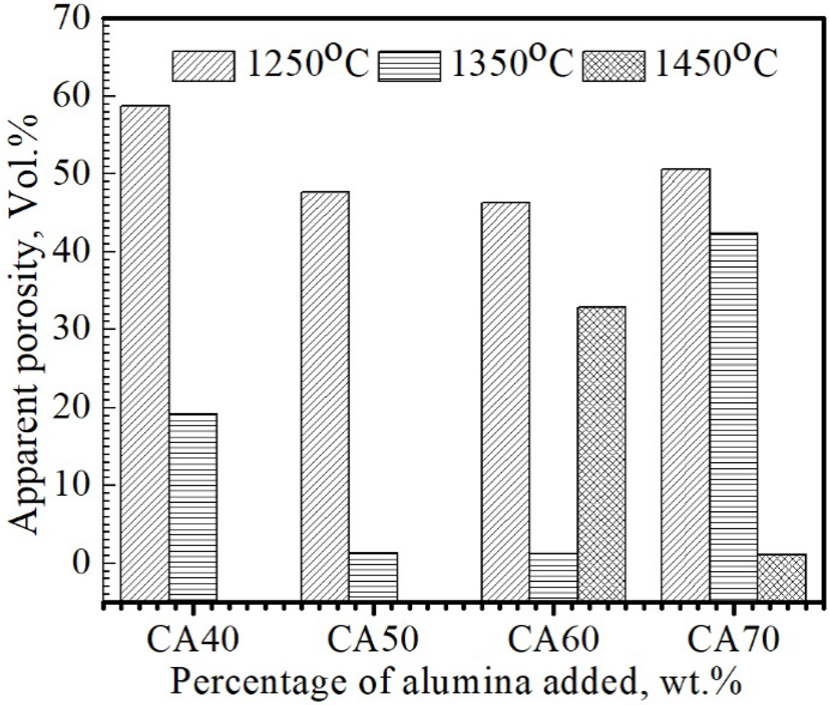
Apparent porosity of the compacted specimens sintered at 1250, 1350 °C and 1450 °C.

**Figure 5:**
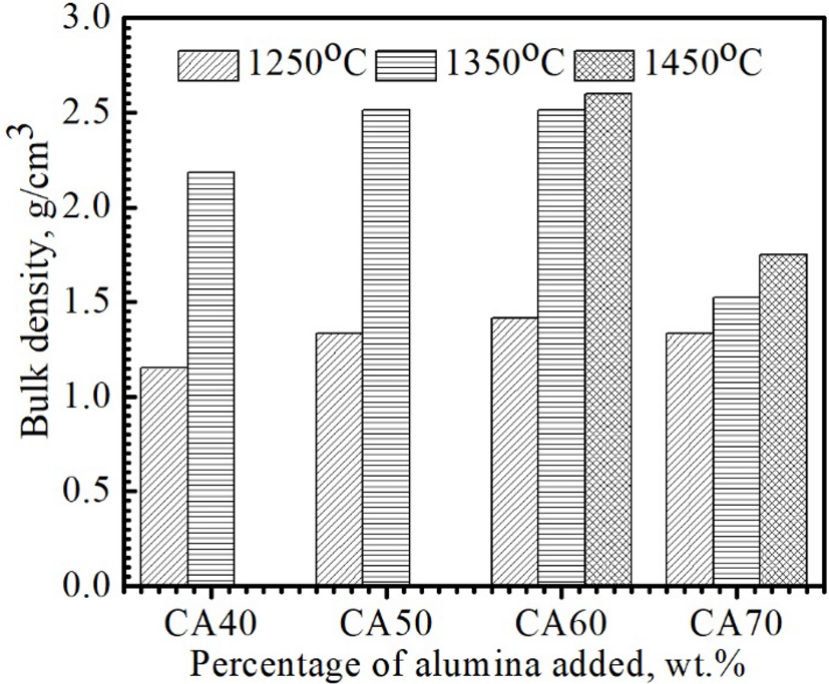
Bulk density of the compacted specimens sintered at 1250, 1350 °C and 1450 °C.

The densification parameters of the sintered specimens “CA40-70” were tested in terms of bulk density and apparent porosity in an attempt to determine the optimal sintering temperature and stoichiometric composition; the results were revealed in Figs. 4 and 5. The apparent porosity of the CA40-60 sintered samples decreased (Fig. 4) and conversely the bulk density enhanced (Fig. 5) with the addition of waste alumina due to the higher density of corundum (D = 3.88–3.99 g/cm^3^)^27–29^ relative to that of limestone (D = 2.71 g/cm^3^)^29–32^. In contrast, bulk density declined and apparent porosity increased unusually with the increase in alumina content beyond CA60. The poor densification characteristics of “CA70” specimens sintered in the temperature range 1250–1450 °C can be explained in terms of the evolution of the high-melting CA_2_ phase in these samples (m.p. = 1750–1770 °C)^33^ at the expense of the low-melting mayenite phase (m.p. = 1390 °C)^34–37^ formed in “CA60” specimens. Regarding the effect of sintering temperature on the densification behavior of sintered samples “CA40-70”, there was an increase in bulk density and a decrease in apparent porosity with the enhancement of sintering temperature from 1250 to 1450 °C due to the phenomenon of sintering densification and the gradual sedimentation of the formed phases in the voids, which reduces the apparent porosity and enhances the bulk density^12,29,38–40^. Among the tested samples, the “CA60” specimen sintered at 1450 °C revealed the lowest apparent porosity (0.9 vol%) and the highest bulk density (2.7 g/cm^3^), revealing a semi-molten, fully-dense structure. This is regarded as the ideal structure for the synthesis of calcium aluminate cement due to the absence of unreacted lime. Unreacted lime has a detrimental effect on refractories because of its high sensitivity to hydration and carbonation. Analysis of the phase composition and densification parameters of the sintered blends “CA40-70” has shown the high prominence of the “CA60” mix for the synthesis of high-quality calcium aluminate cement with fast-setting properties at low firing temperature (1450 °C).

#### Compression resistance

**Figure 6:**
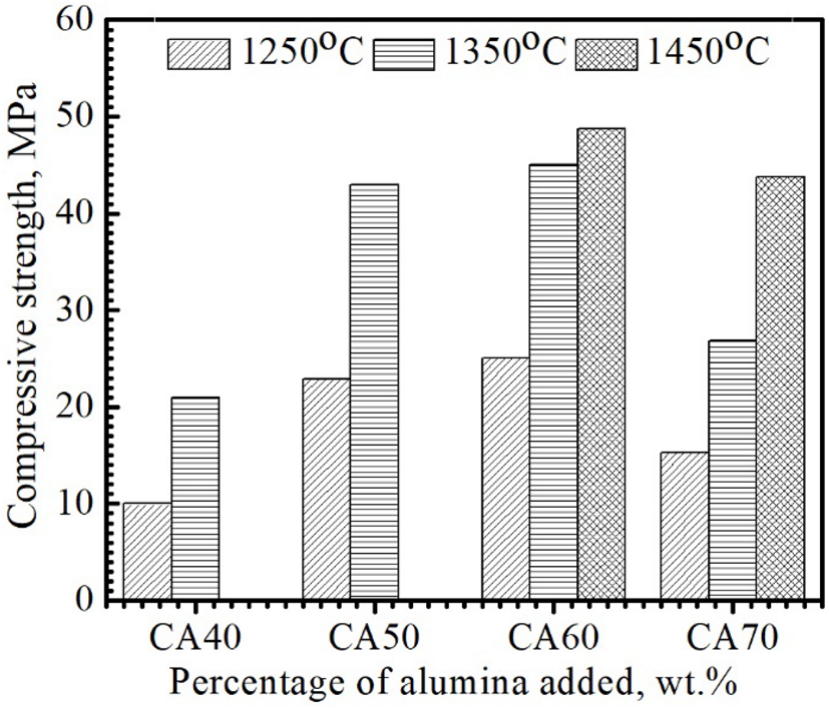
Compreesive strength of the compacted specimens sintered at 1250, 1350 °C and 1450 °C.

Variations in the crushing strength of sintered samples “CA40-70” with sintering temperature and alumina addition were shown in Fig. 6. There was high compatibility between densification behavior and compression resistance values where the compression resistance increased as porosity decreased and density increased by increasing both sintering temperature and alumina content. For the sintered specimen “CA70”, its compression resistance values were slightly lower than those of specimen “CA60”, which was consistent with the enhanced porosity and reduced density of the “CA70” specimen compared to the “CA60” specimen. The highest compression resistance value (49 MPa) was recorded for the “CA60” specimen sintered at 1450 °C because of its entirely dense structure and low porosity. Based on the chemical and physicomechanical characteristics of the “CA40-70” mixes sintered at 1250–1450 °C, the CA60 specimen prepared at 1450 °C was selected to be investigated as a high-strength, fast-setting calcium aluminate cement for military and marine applications. The cementing properties of the “CA60” mix sintered at 1450 °C and screened via a 400-mech sieve were explained in the following sections.

### Hardened samples

#### Phase composition of the hydrated cement and mechanism

**Figure 7:**
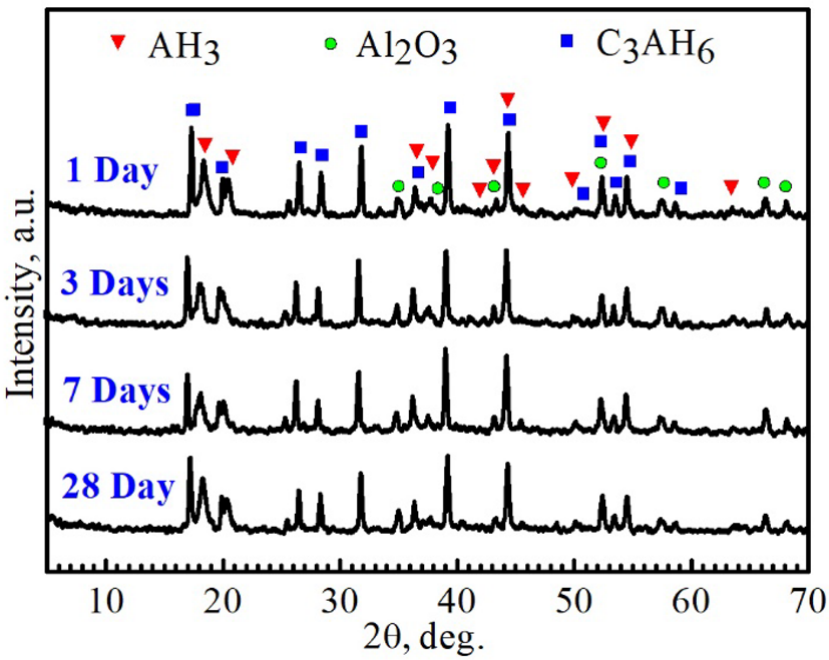
Powder X-ray diffraction patterns of CA60 cement samples hydrated for 1, 3, 7, and 28 days.

Figure 7 demonstrates powder XRD patterns of the CA60 mixes hydrated at 1, 3, 7, and 28 days. As shown in Fig. 7, the hydraulic CA phase reacted with mixing water giving rise to C_3_AH_6_ (Katoite) and AH_3_ (Gibbsite) hydrates, along with trace amounts of Al_2_O_3_ (Corundum)^41^. As calcium aluminate cement CAC (or monocalcium aluminate CA) is placed in water, a solution is formed by the dissolving of calcium ions (Ca^2+^) and aluminate ions (Al(OH)_4_^−^) in the water. Several hydrate types such as C_2_AH_8_, CAH_10_, C_3_AH_6_, and AH_3_ can be formed by the combination of Ca^2+^ and Al(OH)_4_^−^ ions (Eqs. (1)–(4)). At a temperature between 10 and 27 °C and with the presence of C_12_A_7_ as appears in Eq. (5)^42^, the two hydrated phases C_2_AH_8_ and CAH_10_ can present together^41,43^. These phases are metastable, have hexagonal morphology, and their lower energy than the starting phase assemblage provides a driving force for their formation. On the other hand, their energy is higher than the stable phase assemblage, thus these metastable phases react to give the stable phase assemblage. Hence, the subsequent conversion reactions of these metastable phases can form the stable phases C_3_AH_6_ and AH_3_^44^.

1 $${\text{CA }} + { 1}0{\text{H}} \Rightarrow {\text{CAH}}_{{{1}0}} ,$$

2 $${\text{2CA }} + {\text{ 16H}} \Rightarrow {\text{C}}_{{2}} {\text{AH}}_{{8}} + {\text{ AH}}_{{3}} ,$$

3 $${\text{2CAH}}_{{{1}0}} \Rightarrow {\text{C}}_{{2}} {\text{AH}}_{{8}} + {\text{ AH}}_{{3}} + {\text{ 9H}},$$

4 $${\text{3C}}_{{2}} {\text{AH}}_{{8}} \Rightarrow {\text{2C}}_{{3}} {\text{AH}}_{{6}} + {\text{ AH}}_{{3}} + {\text{ 9H}},$$

5 $${\text{C}}_{{{12}}} {\text{A}}_{{7}} + {\text{ 51 H}}_{{2}} {\text{O }} \Rightarrow {\text{ 6 C}}_{{2}} {\text{AH}}_{{8}} + {\text{ AH}}_{{3}} .$$

#### Microstructure of the hydrated samples

**Figure 8:**
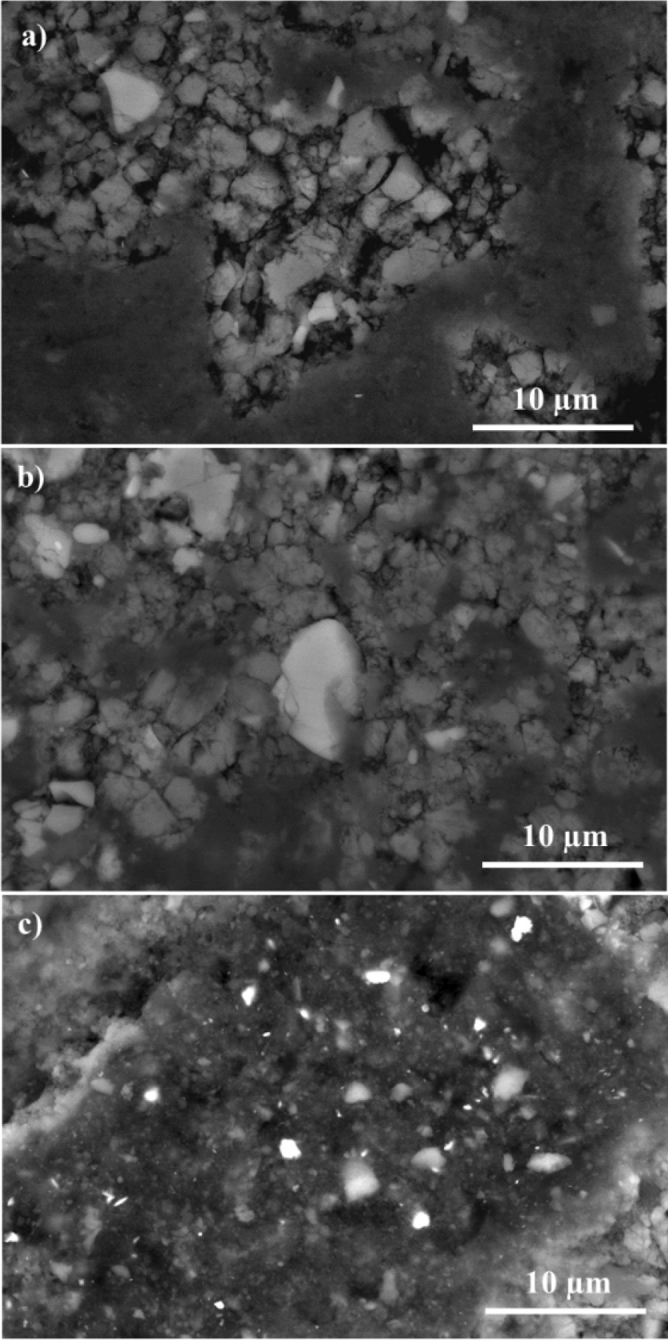
FESEM photomicrographs of the hydrated CA60 cement samples fired at 1450 °C; (**a**) after 1 day, (**b**) after 3 days, (**c**) after 7 days.

The backscattered electron (BSE) field-emission scanning electron microscope (FESEM) has been applied to illustrate the behavior of CAC during different periods of hydration. Figure 8 demonstrates the microstructure of the hydrated calcium aluminate cement CA60 after 1, 3, and 7 days of hydration. A dominated semi-cubic particles appeared clearly with a light grey color in Fig. 8a after 1 day of hydration. These particles are representing C_3_AH_6_ crystals which have a cubic crystal structure and resulted from the hydration of CA to the metastable phases CAH_10_ and C_2_H_8_ which subsequently converted to the stable phase C_3_AH_6_ and AH_3_^44,45^. The C_3_AH_6_ crystals are partially covered with a darker cloud that is attributed to AH_3_ which has a formless mass^44^. In addition, a spaced distribution of the particles can be noticed clearly with significant pores between the particles. After 3 days of hydration as appeared in Fig. 8b, the crystals are densely packed together with the distribution of the hydrated alumina (AH_3_) that gave the particles a darker grey color^40^. Moreover, the size of the crystals has decreased with a significant decrease in the pores as well. Almost full coalescence of the crystals has appeared after 7 days of hydration at Fig. 8c, where the size of the particles has decreased and appeared as small fragments stacked together. It can be concluded that the increase in hydration time has a major effect on the adhesion of crystals. Hence, the cohesion of C_3_AH_6_ crystals along with AH_3_ has the main role in developing a high crushing strength of CAC, since the resulted cement is featured with high density and low porosity between particles.

#### Densification parameters of hydrated cements

**Figure 9:**
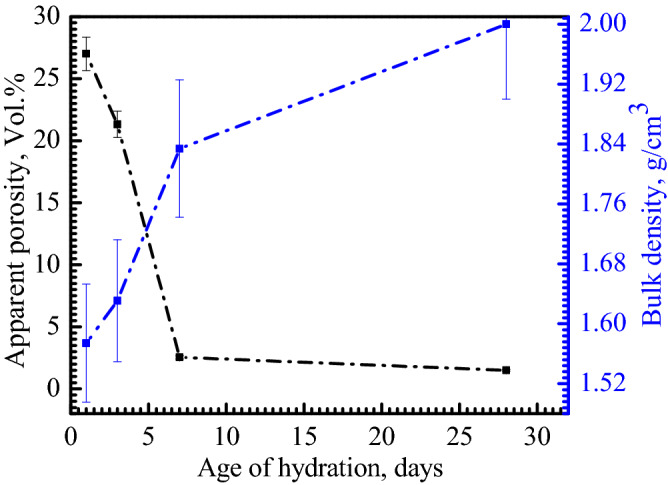
Bulk density and apparent porosity of the hydrated CA60 cement samples sintered at 1450 °C after (1, 3, 7, and 28 days).

The apparent porosity and bulk density of the hydrated cement samples of mix CA60 with 60 wt% Al_2_O_3_ sintered at 1450 °C were investigated. Figure 9 shows the densification parameters after 1, 3, 7, and 28 days of hydration. The apparent porosity of the hydrated samples has decreased upon increasing the hydration days where the minimum porosity (1.5%) occurred after 28 days of hydration. For bulk density, an opposite behavior has taken place where the bulk density increased by increasing the days of hydration until reaches its maxima of 2 g/cm^3^ after 28 days. The reduction in porosity which is accompanied by an increase in density can be explained by the CAC hydration mechanism. The hydrate phases AH_3_, C_3_AH_6_, CAH_10_, and C_2_AH_8_ are precipitated from a saturated solution with a low water/cement ratio. These hydrates are presenting an interlocking effect after chemical transformation and form new bonds which provide low porosity and hence high density^46^.

### Cementing properties

Based on the above results, the optimum composition of the manufactured cement was chosen to be CA60 at an optimum firing temperature of 1450 °C. The cementing properties were applied for the manufactured cement of mix CA60 including the water of consistency, setting time, and crushing strength over different curing times (1, 3, 7, and 28 days).

#### Water of consistency and setting time

**Figure 10:**
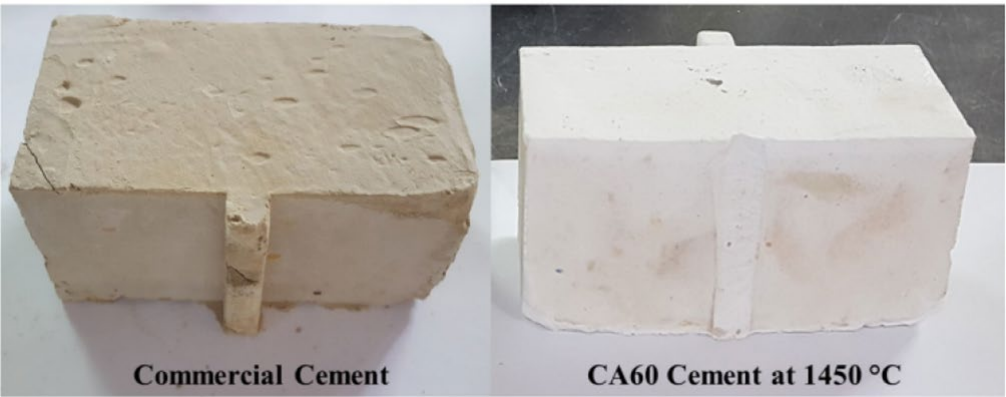
Manufactured CA60 sintered at 1450 °C and commercial cement pastes.

As the main purpose of this research is to manufacture CAC from wastes that are compared to the commercial one; the cementing properties, namely; water of consistency and setting time were compared to commercial calcium aluminate cement. Figure 10 shows the prepared cement with CA60 mix at 1450 °C and commercial cement. Table 4 illustrates the water of consistency and the setting time for the manufactured CA60 and the commercial CAC. The cement paste prepared from mix CA60 has consumed almost the same amount of water as the commercial one. The initial setting time was approximately 45 min and the final setting time was about 135 min whereas the initial and final setting time for the commercial CAC was 30 and 360 min, respectively. This fast setting time of the prepared CAC allows it to be implemented in many applications which required a fast setting time as military applications.

**Table 4:** Water of consistency and setting time of the manufactured and commercial cements.

Cement type	Water of consistency (%)	Setting time (min)	
		Initial	Final
CA60	20.8	45	135
Commercial CAC	20	30	360

#### Mechanical properties of the hydrated cements

**Figure 11:**
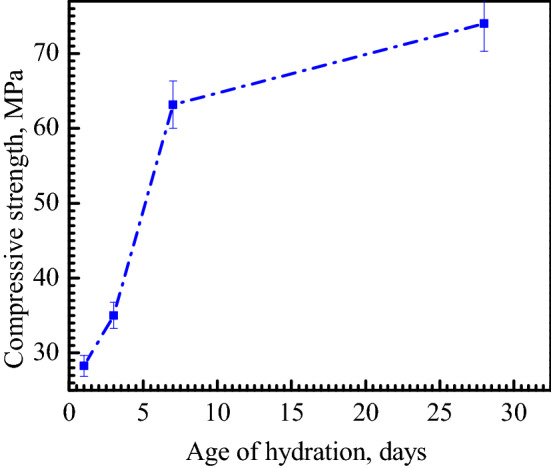
Cold crushing strength of the hydrated CA60 cement samples sintered at 1450 °C after 1, 3, 7, and 28 days of hydration.

Figure 11 represents the cold crushing strength of hydrated cement of mix CA60 for 1, 3, 7, and 28 days. The strength of the hydrated cement sintered at 1450 °C was increased with increasing the days of hydration where after 7 days the strength reaches 63.1 MPa and after 28 days reaches 74 MPa. The high strength of the hydrated cement is related to the presence of CA and C_12_A_7_ phases as main compounds which react rapidly with water^36^. The reaction of CA and C_12_A_7_ occurs at early stages and the exothermic hydration of C_12_A_7_ results in the early formation of stable hydrates which in turn develop high strength at early ages^8,9^.

### Unshaped refractory castable characteristics

**Figure 12:**
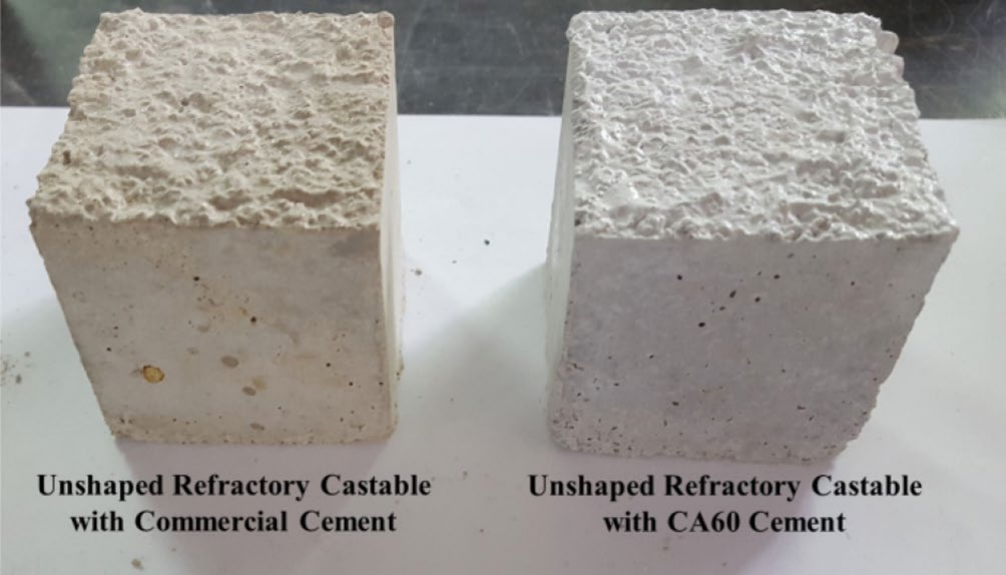
Unshaped refractory castables using CA60 sintered at 1450 °C and commercial cement.

**Figure 13:**
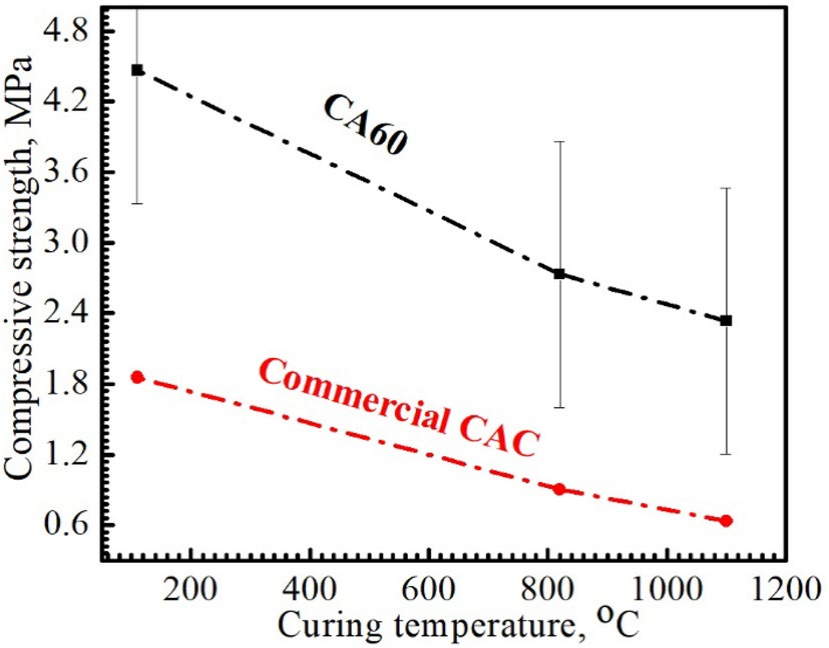
Effect of curing temperature at 110, 820, and 1100 °C, on the crushing strength of castables.

Conventional castables (5 × 5 × 5 cm^3^) were prepared from mixtures composed of 15 wt% cement and 85 wt% aggregates (40% Al_2_O_3_), where CA60 and commercial cement were used to compare the effect of the manufactured CA60 cement with the commercial one. The prepared castables appear in Fig. 12 and have been cured at different temperatures (110, 820, and 1100 °C) for 2 h. The crushing strength in Fig. 13, illustrates the descending in strength upon increasing the curing temperature for the prepared castables with both cements. The castables prepared with CA60 cement have shown a higher strength at 110 °C with 4.5 MPa when compared to the commercial CAC at the same temperature (1.8 MPa). With further increase in curing temperature, the strength of the castables has collapsed due to the consequence of evaporation of water as the temperature increased; followed by extra creation of pores and lower strength of the refractory castables^29,47^. This huge difference in strength between CA60 and commercial cement has promoted and stated the industrial and economical value of the manufactured CA60 cement, where a higher strength castables with fast-setting time can be achieved by applying prepared cement from waste instead of commercial cement.

## Concluded remarks

1. High-strength calcium aluminate cement with high purity was successfully prepared from industrial waste that contains high alumina content (97.5%) and limestone contains 98.6% of CaO.
2. A simple sintering method at different temperatures of 1250, 1350, and 1450 ℃ was applied for four mixtures that have different alumina content from 40 to 70 wt%. The optimum sintering temperature selected was 1450 ℃ and the optimum cement mixture has 60 wt% of alumina. The resulting cement under these optimum conditions has shown the best sintering parameters and achieved high strength reaches to 74 MPa after 28 days of hydration.
3. The prepared CAC and commercial CAC were used in the preparation of castables to investigate their properties in various applications. The castables fabricated with the prepared CAC have shown higher strength than that of the commercial CAC which assures its advantage over cement products.

## Data Availability

The datasets generated during and/or analyzed during the current study are available from the corresponding author on reasonable request.
